# Design of Oscillatory Networks through Post-Translational Control of Network Components

**DOI:** 10.35534/sbe.2023.10004

**Published:** 2023-03-13

**Authors:** Brianna E.K. Jayanthi, Shridhar Jayanthi, Laura Segatori

**Affiliations:** 1 Systems, Synthetic, and Physical Biology Graduate Program, Rice University, Houston, TX 77005, USA; 2 Department of Bioengineering, Rice University, Houston, TX 77005, USA; 3 Department of Chemical & Biomolecular Engineering, Rice University, Houston, TX 77005, USA; 4 Department of BioSciences, Rice University, Houston, TX 77005, USA

**Keywords:** Genetic oscillators, Protein degradation, Post-translational regulation, Nanobody, Synthetic genetic networks

## Abstract

Many essential functions in biological systems, including cell cycle progression and circadian rhythm regulation, are governed by the periodic behaviors of specific molecules. These periodic behaviors arise from the precise arrangement of components in biomolecular networks that generate oscillatory output signals. The dynamic properties of individual components of these networks, such as maturation delays and degradation rates, often play a key role in determining the network’s oscillatory behavior. In this study, we explored the post-translational modulation of network components as a means to generate genetic circuits with oscillatory behaviors and perturb the oscillation features. Specifically, we used the NanoDeg platform—A bifunctional molecule consisting of a target-specific nanobody and a degron tag—to control the degradation rates of the circuit’s components and predicted the effect of NanoDeg-mediated post-translational depletion of a key circuit component on the behavior of a series of proto-oscillating network topologies. We modeled the behavior of two main classes of oscillators, namely relaxation oscillator topologies (the activator-repressor and the Goodwin oscillator) and ring oscillator topologies (repressilators). We identified two main mechanisms by which non-oscillating networks could be induced to oscillate through post-translational modulation of network components: an increase in the separation of timescales of network components and mitigation of the leaky expression of network components. These results are in agreement with previous findings describing the effect of timescale separation and mitigation of leaky expression on oscillatory behaviors. This work thus validates the use of tools to control protein degradation rates as a strategy to modulate existing oscillatory signals and construct oscillatory networks. In addition, this study provides the design rules to implement such an approach based on the control of protein degradation rates using the NanoDeg platform, which does not require genetic manipulation of the network components and can be adapted to virtually any cellular protein. This work also establishes a framework to explore the use of tools for post-translational perturbations of biomolecular networks and generates desired behaviors of the network output.

## 1. Introduction

Many cellular functions are regulated by molecules that exhibit periodicity of expression or activity [1,2]. Oscillatory processes appear in a wide variety of contexts ranging from alternating periodic expression of the CLOCK:BMAL1 and PER:CRY [3] complexes in circadian cycles and the cyclin:CDK and APC:Cdc20 [4] complexes in cell cycle progression to the periodic activity of p53 [5] mediating DNA repair upon cell irradiation. Other examples of oscillatory behaviors that determine cell fate include NF-κB-mediated regulation of the inflammatory response [6], segmentation activity in early embryogenesis [7], neuronal stem cell differentiation [8], and deregulation of circadian cycles associated with Alzheimer’s disease [9]. In all these biological oscillators, the specific arrangement of biomolecular components gives rise to a periodic behavior that is frequently an important determinant of cellular fate. The underlying network properties and dynamic interactions that result in such cell-fate-determining oscillations, however, remain to be fully characterized. The development of tools specially designed to initiate or perturb oscillatory mechanisms and thus control the resulting cellular behavior would open the way to the study of these endogenous networks and provide well-defined modules to build genetic networks and create novel biological functions.

Oscillatory signals are commonly characterized by the amplitude and period of the oscillation and are intrinsically linked to the dynamics of individual nodes of a biomolecular network. Oscillations arise from the presence of a limited cycle around an unstable equilibrium or from an excitable response [10] that presents a very long transient (damped) oscillation. Both the excitable system response and the limited cycles around an unstable equilibrium are associated with systems that present a supercritical Hopf bifurcation for a critical parameter of the system [10–12]. Specifically, limited cycles appear when the value of the critical parameter is such that an equilibrium becomes unstable (i.e., the critical parameter “crosses” the Hopf bifurcation value), whereas the excitable response appears when the value of the critical parameter is such that the equilibrium is still stable but approaches the critical factor (i.e., the critical parameter approaches the Hopf bifurcation value). Dynamic parameters such as delays [13,14] and degradation rates [15–18] are frequently critical parameters in Hopf bifurcations associated with the onset of oscillations in biomolecular networks. Changing these dynamic parameters, however, typically requires genetic engineering of the target protein to modify inherent protein properties, such as maturation or degradation rates—An approach that may require cumbersome protein engineering and is inevitably poised to alter the innate protein function. Target-specific molecules, such as small-molecule inhibitors [5], provide an alternative means to control specific properties of the target protein but present target-specific availability and often limited specificity. There is thus a need for universal tools that can be adapted to control a diverse range of network components and the phenotypic response study to oscillatory behaviors.

Post-translational control of protein properties provides exquisite control over the target protein’s kinetics as post-translational events typically occur over timescales faster than transcriptional and translational events. Targeting proteins at the post-translational level also expands the target specificity to post-translational modifications [19,20], which are frequent determinants of protein function [21]. Post-translational control of proteins can be easily achieved via proteasomal degradation of the target, but typically requires manipulation of the target [22–26] and presents target-specific efficiency [27–29]. Nanobody-mediated targeting of cellular proteins to proteasomal degradation was recently reported as a strategy to achieve exquisite control over proteins’ steady-state levels without genetic manipulation of the target [30]. The NanoDeg platform consists of a heterobifunctional molecule comprising a target-specific nanobody and a degradation sequence (degron). Binding of the degron-tagged nanobody to the target results in proteasomal degradation of the resulting complex with kinetic properties that depend on the degron tag’s sequence. Transcriptional regulation of the NanoDeg also allows adjusting target protein levels [31]. The NanoDeg provides a flexible platform that can be customized to target potentially any cellular protein by evolving a target-specific nanobody and to achieve the desired target’s steady-state levels by altering the degradation tag. Furthermore, NanoDeg-mediated perturbations of genetic networks can be produced through transfection or viral transduction, making the NanoDeg an ideal tool to generate dynamic perturbations through facile cell engineering approaches.

In this work, we explored post-translational regulation of circuit components using the NanoDeg as a strategy to create oscillatory outputs in non-oscillating gene circuits and to modulate the output’s oscillatory features. To achieve this goal, we explored strategies to perturb representative systems from two main classes of biomolecular oscillators, namely relaxation oscillators (the activator-repressor and the Goodwin oscillator) and ring oscillators (repressilators). We simulated relaxation oscillator and ring oscillator topologies upon integration of the NanoDeg with the ultimate goal to obtain design rules for building oscillators through post-translational modulation of circuit components. The first two subsections focus on a two-node activator-repressor topology and a single-node self-repressing Goodwin oscillator topology. The third subsection describes a three-node repressilator topology that is perturbed by the addition of a common NanoDeg targeting all the repressilator nodes or by the addition of multiple node-specific NanoDegs. Finally, in the fourth subsection, we investigate the use of the NanoDeg as one of the repressilator nodes. The results obtained illustrate different approaches to designing and controlling genetic circuits with oscillatory behaviors via post-translational modulation of circuit components achieved by altering the components’ degradation rate.

## 2. Methods

The simulations reported in this work were obtained using MATLAB R2019a, The Mathworks. The activator-repressor system and the two repressilators were simulated using continuous ordinary differential equation (ODE) solver “ode23s,” an order (2, 3) solver modified to work with stiff systems. The Goodwin oscillator was simulated using the DDE solver “dde23,” an order (2, 3) solver. The models we developed for these systems were reduced by using singular perturbation to capture the essential dynamic behavior of the systems studied [32,33].

The amplitude of oscillation was obtained by calculating the difference between the largest and the lowest concentration of the reported protein in the region of oscillation using MATLAB’s “range” function. The period of oscillation was obtained by first subtracting the mean value of concentration in the region of oscillation from the simulated trajectory for the concentration of a reported protein to obtain a zero-mean trajectory. Subsequently, the periods were calculated by averaging the time interval between alternating zero crossings of the zero-mean trajectory in the region of oscillation. A simulation sampling period of 0.01 h was imposed in all simulations to prevent errors in the calculation of periods and amplitudes due to low sampling.

### General Assumptions

The mathematical models built to simulate the behavior of the topologies explored in this study were based on the assumptions that the formation of a complex between the NanoDeg and a transcriptional regulator prevents the interaction between the transcriptional regulator and its cognate operator and that the NanoDeg-target complex is degraded at the same rate as the free NanoDeg. Simulations of the system without NanoDeg were conducted by setting $k_{N}=\;0$, and simulations with a nominal concentration of NanoDeg were conducted by setting $k_{N}=N_{0}\text{/}\delta_{N}$, where $N_{0}$ is the nominal concentration of NanoDeg.

## 3. Results

### 3.1. Activator-Repressor

**Figure 1.**
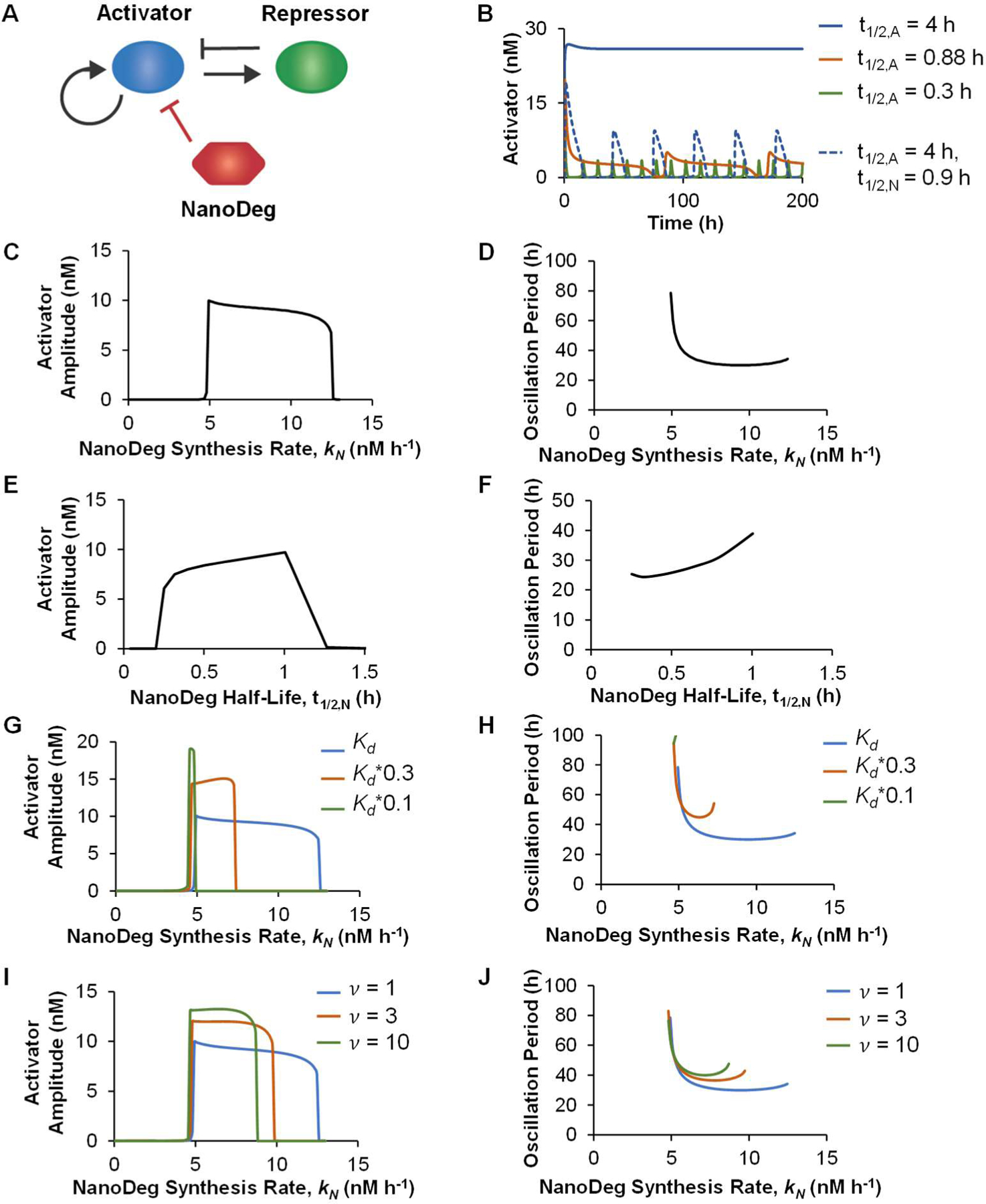
The activator-repressor oscillator. (**a**) Schematic representation of the activator-repressor oscillator with an activator-targeting NanoDeg. (**b**) Concentration of the activator in an activator-repressor circuit with an activator with a half-life $\left(t_{1/2,\text{A}}\right)$ of 4 h (blue), 0.88 h (red), or 0.3 h (green), or with an activator with a half-life of 4 h and an activator-targeting NanoDeg with a half-life $\left(t_{1/2,\text{N}}\right)$ of 0.9 h (dashed blue) as a function of time. (**c,d**) Amplitude (**c**) and period of oscillation (**d**) of the activator in an activator-repressor circuit with an activator-targeting NanoDeg ($t_{1/2,\text{A}}=4\;\text{h}$; $t_{1/2,\text{N}}=0.9\;\text{h}$) as a function of the NanoDeg synthesis rate. (**e,f**) Amplitude (**e**) and period of oscillation (**f**) of the activator in an activator-repressor circuit with an activator-targeting NanoDeg ($t_{1/2,\text{A}}=4\;\text{h}$) as a function of the NanoDeg half-life. (**g,h**) Amplitude (**g**) and period of oscillation (**h**) of the activator in an activator-repressor circuit with an activator-targeting NanoDeg ($t_{1/2,\text{A}}=4\;\text{h}$; $t_{1/2,\text{N}}=0.9\;\text{h}$) as a function of the NanoDeg synthesis rate and NanoDeg-activator equilibrium dissociation constants ($K_{d}=4.41\;\text{nM}$, blue; 1.47 nM, red; or 0.441 nM, green). (**i,j**) Amplitude (**i**) and period of oscillation (**j**) of the activator in an activator-repressor circuit with an activator-targeting NanoDeg ($t_{1/2,\text{A}}=4\;\text{h}$; $t_{1/2,\text{N}}=0.9\;\text{h}$) as a function of the NanoDeg synthesis rate, with constant $K_{d}$ of 4.41 nM, and with association and dissociation rate constants ($k_{on}$ and $k_{off}$) scaled by a common factor (v=1, blue; 3, red; or 10, green). The amplitude of oscillation was obtained by calculating the difference between the largest and lowest concentration of the activator in the region of oscillation using MATLAB’s “range” function. The period of oscillation was calculated by averaging the time interval between alternating zero crossings of the zero-mean trajectory in the region of oscillation.

Activator-repressor systems are two-node genetic circuits that present a Hopf bifurcation, wherein a stable equilibrium point bifurcates into an unstable equilibrium and a stable periodic orbit upon an increase in the separation of timescales between activator and repressor dynamics [15,34]. The fundamental mechanism responsible for this transition is well captured by a two-dimensional model that describes the rate of change of the activator and repressor concentrations [15]. The activator-repressor topology considered here consists of a transcriptional activator that activates its own expression as well as that of a transcriptional repressor that represses the expression of the activator (Figure 1A). To explore the effect of post-translational regulation on the behavior of the activator-repressor topology, we first built a model based on ordinary differential equations describing the concentration of the activator, the repressor, and an activator-specific NanoDeg. The concentrations of all species were derived as dependent on the rate of synthesis and rate of degradation, with the rates of synthesis modeled as constitutive or following Hill functions for an activator or a repressor [35] and the rates of degradation either reflecting the innate protein’s half-life or the half-life of the NanoDeg. The interaction between the NanoDeg and its target was modeled by mass-action expressions.

The following differential equations were used to simulate the expression of the activator-repressor system’s components:

(1) $$\frac{\text{d}A}{\;\text{d}t}=p_{A_{T}}\frac{\left(\alpha_{1}A^{m}\;+\;\alpha_{2}K_{A}^{m}\right)\left(\beta_{1}B^{n}\;+\;\beta_{2}K_{B}^{n}\right)}{\left(A^{m}\;+\;K_{A}^{m}\right)\left(B^{n}\;+\;K_{B}^{n}\right)}-\delta_{A}A-k_{on}AN\;+\;k_{off}C$$

(2) $$\frac{\text{d}B}{\;\text{d}t}=p_{B_{T}}\frac{k_{5}A^{m}\;+\;k_{6}K_{A}^{m}}{A^{m}\;+\;K_{A}^{m}}-\delta_{B}B$$

(3) $$\frac{\text{d}N}{\;\text{d}t}=p_{N_{T}}k_{N}-\delta_{N}N-k_{on}AN+k_{off}C$$

(4) $$\frac{\text{d}C}{\;\text{d}t}=k_{on}AN-k_{off}C-\delta_{N}C$$

where A is the concentration of the unbound activator, B is the concentration of the unbound repressor, N is the concentration of the unbound NanoDeg, and C is the concentration of the NanoDeg-activator complex. The expression of A was modeled as dependent on the rate of synthesis regulated by A and B with independent binding following a multiplicative model that combines a Hill Function for self-activation due to A and for repression due to B. Self-activation of A depends on the Hill coefficient (m), the maximum rate of synthesis due to self-activation $\left(\alpha_{1}\right)$, the rate of synthesis due to leakiness $\left(\alpha_{2}\right)$, with the requirement that $\alpha_{1\;>\;}\alpha_{2}$, and the equilibrium dissociation constant of A binding to its operator sequence $\left(K_{A}\right)$. Repression of A due to B depends on the Hill coefficient $\left(\text{n}\right)$, the minimum rate of synthesis due to repression $\left(\beta_{1}\right)$, the rate of synthesis due to leakiness $\left(\beta_{2}\right)$, with the requirement that $\beta_{2}>\beta_{1}$, and the equilibrium dissociation constant of B binding to its operator sequence $\left(K_{B}\right)$. The concentration profile of A is also dependent on a linear degradation rate $\left(\delta_{A}\right)$ and association and dissociation of the NanoDeg and activator governed by the rate constants $k_{on}$ and $k_{off}$. The expression of B was modeled as dependent on a linear degradation rate $\left(\delta_{B}\right)$ and rate of synthesis regulated by A following a Hill function with the Hill coefficient (m), the maximum rate of synthesis $\left(k_{5}\right)$, the rate of synthesis due to leakiness $\left(k_{6}\right)$, and the equilibrium dissociation constant of A binding to its operator sequence $\left(K_{A}\right)$. The constitutive expression of NanoDeg was modeled as dependent on the rates of synthesis $\left(k_{N}\right)$ and degradation $\left(\delta_{N}\right)$ and the association and dissociation of the NanoDeg and activator governed by the rate constants $k_{on}$ and $k_{off}$. The simulations were conducted using the parameter values reported in Table S1 of the Method Details unless the otherwise specified.

The onset of output oscillation requires a separation of timescales between the activator and repressor dynamics, which is achieved when the synthesis and degradation of the activator are greater than that of the repressor [15,18,34]. We first investigated the use of the NanoDeg for triggering oscillatory behaviors in an activator-repressor system that did not exhibit oscillation due to the lack of the separation of timescales between the activator and repressor dynamics. Specifically, we modeled an activator-repressor topology with activator and repressor proteins exhibiting equal half-lives (i.e., degradation rates). We then introduced a NanoDeg that bond specifically to the activator and modulates the activator’s degradation rate through NanoDeg-mediated post-translational depletion (Figure 1A). Such an activator-specific NanoDeg could be generated using an activator-specific nanobody [36,37] or using a fluorescent protein- [38] or peptide tag-specific [39] nanobody upon co-expression of the activator appropriately engineered by fusion to the fluorescent protein or peptide tag. The activator-repressor output was modeled based on a short half-life NanoDeg $\left(t_{½}=0.9\;\text{h}\right)$, which was experimentally demonstrated to result in the greatest reduction in the steady-state levels of a stable target protein [30].

Simulation of the activator-repressor circuit based on both the activator and the repressor exhibiting a half-life of 4 h generated a non-oscillating output (Figure 1B, blue). As is expected, the system rapidly reaches a stable equilibrium due to the activator and repressor operating at similar timescales [15,34]. Decreasing the half-life of the activator to generate a timescale separation results in onset of oscillations. For the chosen parameters, oscillation is triggered by lowering the half-life of the activator to at least 0.88 h (Figure 1B, red), with shorter activator half-lives increasing the frequency of oscillation (Figure 1B, green). Such a drastic alteration in a protein’s half-life would be challenging to achieve experimentally, as it requires substantial modification of inherent protein properties, including fusion to protease-sensitive tags or protein engineering to alter protein stability. The separation of timescales required for oscillation, however, could be easily achieved through the co-expression of an activator-specific NanoDeg. Because the introduction of the NanoDeg into the system is expected to enhance the degradation rate of the target proportionally to the NanoDeg half-life [30], oscillation of the activator-repressor circuit can be triggered using an activator-specific NanoDeg exhibiting a sufficiently short half-life (Figure 1B, dashed blue). Notably, while introducing a NanoDeg with a half-life of 0.9 h results in an oscillatory output, the same activator-repressor circuit based on an activator with a half-life of 0.9 h in the absence of the NanoDeg does not present oscillatory behavior. This result can be attributed to sequestration effects [40–42] as the model is based on the assumption that the complex between the activator and the NanoDeg cannot be bond to the activator’s cognate promoter [37].

To investigate the extent to which the properties of the NanoDeg influence the design of an activator-repressor circuit in which the output’s oscillatory behavior depends on NanoDeg-mediated control of the activator half-life, we first evaluated the circuit’s output upon modulation of the NanoDeg synthesis rate $\left(k_{N}\right)$ and the NanoDeg half-life $\left(t_{1/2,\text{N}}\right)$. Modulation of the NanoDeg synthesis rate revealed the range of NanoDeg synthesis rates that results in an oscillatory output ($k_{N}=4.9–12.5$, Figure 1C,D). Neither the oscillation amplitude (Figure 1C) nor the oscillation period (Figure 1D), however, was found to vary dramatically upon modulation of the NanoDeg synthesis rate within this range, except for a sharp decline in oscillation period at low NanoDeg synthesis rates. The lower bound of the NanoDeg synthesis rate interval corresponds to the minimum NanoDeg synthesis rate needed to sufficiently enhance the degradation of the activator with respect to that of the repressor. Increasing the NanoDeg synthesis rate above the upper bound results in excessive degradation of the activator so that the activator never reaches a concentration above the threshold needed to activate the expression of the repressor. The amplitude of oscillation decreases moderately in response to an increase in the NanoDeg synthesis rate (Figure 1C). The moderate decrease in amplitude and the robust period (Figure 1D) observed in a large region of NanoDeg synthesis rates suggest that the addition of the NanoDeg to the system triggers oscillations but modulating the NanoDeg synthesis rate within the oscillation-inducing range does not affect the oscillatory behavior of the circuit.

Modulating the NanoDeg half-life revealed that the period of oscillation in the activator-repressor system is sensitive to the NanoDeg half-life. Increasing the NanoDeg half-life results in a moderate increase in the oscillation amplitude (Figure 1E) and a substantial increase in the oscillation period (Figure 1F). Compared to the NanoDeg synthesis rate, modulating the NanoDeg half-life has a more pronounced effect on the period of oscillation within the range of NanoDeg half-lives where oscillation occurs $(t_{1/2,\text{N}}=0.3–1\text{ h)}$ (Figure 1F). This result suggests modulation of the NanoDeg half-life as a potential strategy to control the period of oscillations independently of the amplitude.

To investigate the effect of the kinetics of the interaction between the activator and the NanoDeg on the circuit oscillatory behavior, we modulated the equilibrium dissociation constant $\left(K_{d}\right)$ and the rate constants of association and dissociation ($k_{on}$ and $k_{off}$) governing the interaction between the NanoDeg and the activator and evaluated the oscillation amplitude and the oscillation period as a function of NanoDeg synthesis rate (Figure 1G–J). Decreasing the $K_{d}$ of the interaction between the NanoDeg and the activator, simulated by increasing $k_{on}$, increased the oscillation amplitude (Figure 1G) and the oscillation period (Figure 1H). The range of NanoDeg synthesis rates that result in oscillation is reduced proportionally to the decrease in $K_{d}$ (e.g., decreasing $K_{d}$ by a factor of 10 reduces the region of oscillation to approximately one-tenth of the initial range). The minimum NanoDeg synthesis rate required to trigger oscillation is also slightly reduced with a decrease in the $K_{d}$ (Figure 1G,H). Increasing the rate constants of the interaction between the NanoDeg and the activator by introducing a common scaling factor (v) to increase $k_{on}\left(v\cdot k_{on}\right)$ and $k_{off}\left(v\cdot k_{off}\right)$ and maintain $K_{d}$ constant results in an increase in oscillation amplitude (Figure 1I) and oscillation period (Figure 1J). Similar to the effect observed upon reduction of $K_{d}$, increasing the association and dissociation rate constants of the NanoDeg and the activator causes a slight reduction in the minimum NanoDeg synthesis rate needed to trigger oscillation and a reduction in the range of NanoDeg synthesis rate that results in oscillations. The effect of varying the binding and dissociation rate constants of the NanoDeg and the activator on oscillation amplitude, however, is non-linear (Figure 1I,J). These results indicate that both the affinity and rate constants of the interaction between the NanoDeg and the activator influence the oscillatory behavior of the activator-repressor circuit and are relevant parameters to consider for experimental implementation.

Taken together, these results provide design rules for building an activator-repressor circuit that oscillates upon the expression of an activator-specific NanoDeg. Specifically, the NanoDeg can generate activator-repressor oscillators from activator-repressor systems that do not oscillate due to a lack of timescale separation. Furthermore, the oscillation amplitude does not depend on the NanoDeg synthesis rate and NanoDeg half-life within the oscillation-inducing ranges of NanoDeg synthesis and degradation rate. The period of oscillation, however, is sensitive to variations in the NanoDeg half-life, pointing to a mechanism for modulating the oscillation period independent of the oscillation amplitude. These results also demonstrate that the rates of interaction and the affinity between the NanoDeg and the activator affect the output oscillatory behavior.

### 3.2. Goodwin Oscillator

**Figure 2.**
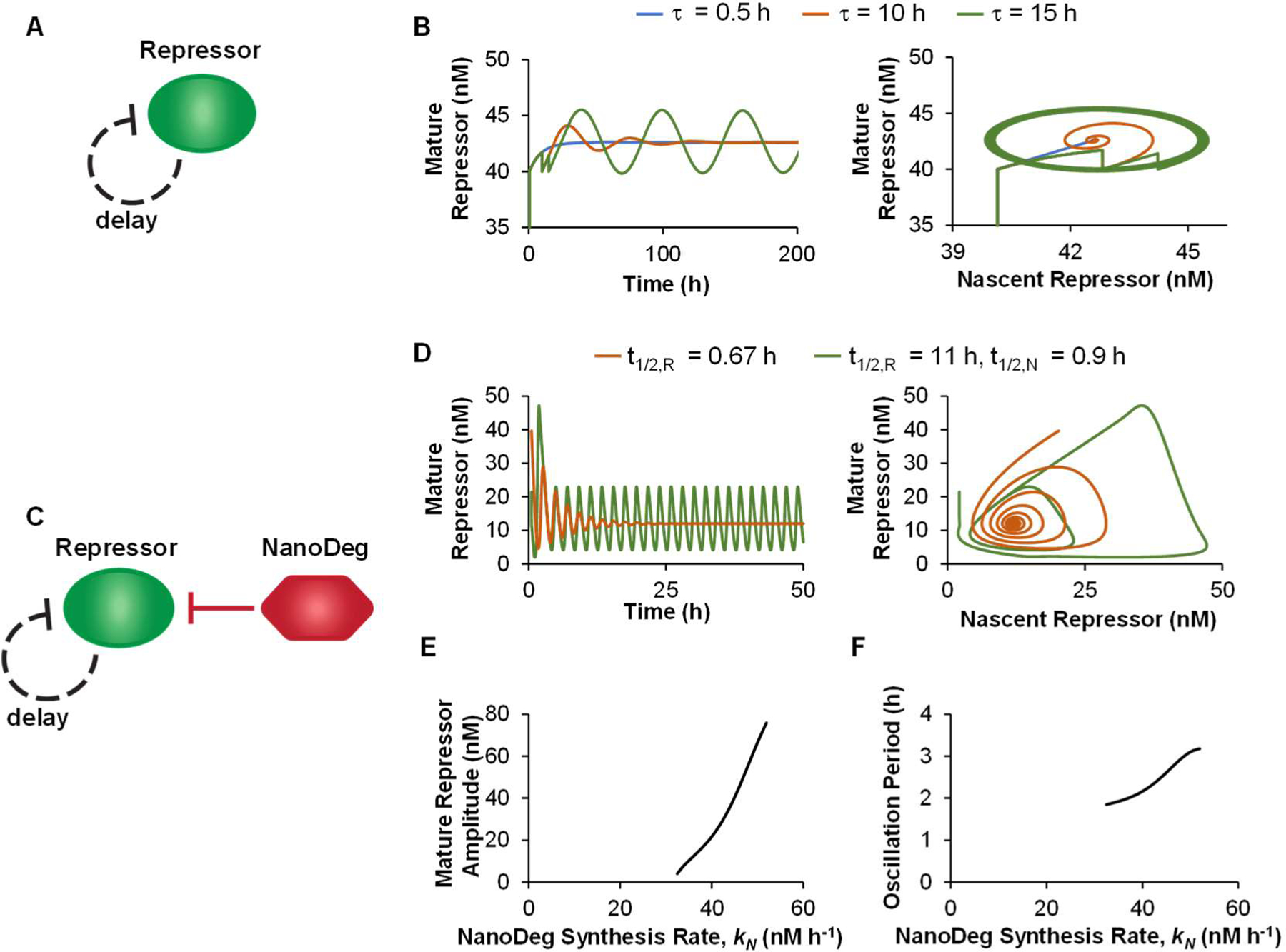
The Goodwin oscillator. (**a**) Schematic representation of the single-gene Goodwin oscillator. (**b**) Concentration of mature repressor as a function of time (left) and as a function of nascent repressor concentration (right) in a Goodwin oscillator with a maturation delay (τ) of 0.5 h (blue), 10 h (red), or 15 h (green) and with a mature repressor with a half-life $\left(t_{1/2,\text{R}}\right)$ of 11 h. (**c**) Schematic representation of the Goodwin oscillator with a mature repressor-targeting NanoDeg. (**d**) Concentration of the mature repressor in a Goodwin oscillator with a maturation delay (τ) of 0.5 h and with a mature repressor with a half-life $\left(t_{1/2,\text{R}}\right)$ of 0.67 h (red) or with a mature repressor with a half-life of 11 h and a mature repressor-targeting NanoDeg with a half-life $\left(t_{1/2,\text{N}}\right)$ of 0.9 h (green) as a function of time (left) and as a function of nascent repressor concentration (right). (**e,f**) Amplitude (**e**) and period of oscillation (**f**) of a Goodwin oscillator with a maturation delay (τ) of 0.5 h, a mature repressor with a half-life $\left(t_{1/2,\text{R}}\right)$ of 11 h, and a Mature Repressor-targeting NanoDeg with a half-life $\left(t_{1/2,\text{N}}\right)$ of 0.9 h as a function of the NanoDeg synthesis rate $\left(k_{N}\right)$. The amplitude of oscillation was obtained by calculating the difference between the largest and lowest concentration of the repressor in the region of oscillation using MATLAB’s “range” function. The period of oscillation was calculated by averaging the time interval between alternating zero crossings of the zero-mean trajectory in the region of oscillation.

Goodwin oscillators are single-protein networks consisting of a protein repressing its own expression and presenting a delay in maturation which causes a repressive effect [43]. The delay in protein maturation introduces a lag between the protein’s expression and its function as a transcriptional repressor [44,45]. When the maturation delay is sufficiently long, the system oscillates between periods characterized by the accumulation of inactive protein and periods characterized by repression of protein expression due to the accumulation of nascent protein during the lag interval, resulting in oscillation in the concentration of active repressor protein. The oscillatory behavior in the Goodwin topology arises from the presence of a supercritical Hopf bifurcation associated with the repressor maturation delay [12,13]. If the maturation delay exceeds the critical Hopf bifurcation value, the oscillation appears as a limit cycle around an unstable equilibrium. If the maturation delay approaches but does not exceed the critical value, a transient oscillation towards a stable equilibrium occurs. The Goodwin topology (Figure 2A) was modeled using a delay-differential equation (DDE) describing the concentrations of a repressor in both nascent and mature forms and assuming that the nascent repressor does not interact with its cognate operator.

The Goodwin topology was modeled using the following equations:

(5) $$\frac{\text{d}A}{\;\text{d}t}=p_{A_{T}}\frac{\beta_{1}A_{\tau }^{m}\;+\;\beta_{2}K_{A}^{m}}{A_{\tau }^{m}\;+\;K_{A}^{m}}-\delta_{A}A-k_{on}A_{\tau }N\;+\;k_{off}C$$

(6) $$\frac{\text{d}N}{\;\text{d}t}=p_{N_{T}}k_{N}-\delta_{N}N-k_{on}A_{\tau }N+k_{off}C$$

(7) $$\frac{\text{d}C}{\;\text{d}t}=k_{on}A_{\tau }N-k_{off}C-\delta_{N}C$$

where $A=A\left(t\right)$ is the free nascent repressor, $A_{\tau }=A\left(t-\tau \right)$ is the mature repressor, τ is the maturation delay, N is the free NanoDeg, and C is the complex that forms upon association of the NanoDeg and the mature repressor $A_{\tau }$. The Goodwin oscillator simulations were initialized by setting $A\left(t\right)=0$ for −τ≤t≤0. Expression of the repressor is simulated using a Hill function for repression by the mature repressor $A_{\tau }$ dependent on the Hill coefficient $\left(\text{m}\right)$, the minimum rate of synthesis due to repression $\left(\beta_{1}\right)$, the rate of synthesis due to leakiness $\left(\beta_{2}\right)$, and the equilibrium dissociation constant of $A_{\tau }$ binding to its operator sequence $\left(K_{A}\right)$. The concentration profile of the mature repressor is also dependent on a linear degradation rate $\left(\delta_{A}\right)$ and the association and dissociation rates of the NanoDeg and $A_{\tau }$ governed by the rate constants $k_{on}$ and $k_{off}$. Constitutive expression of the NanoDeg is simulated using a constant synthesis rate $\left(k_{N}\right)$ and a linear degradation rate $\left(\delta_{N}\right)$. The association and dissociation interactions between the mature repressor $A_{\tau }$ and the NanoDeg are modeled using a mass-action reaction model. The simulation was conducted using the parameter values reported in Table S2 of the Methods Details unless the otherwise specified.

Simulation of the Goodwin topology with a repressor half-life $\left(t_{1/2,\text{R}}\right)$ of 11 h and a repressor maturation delay (τ) of 0.5 h exhibits a stable equilibrium (Figure 2B, blue). Increasing the maturation delay $\left(\text{τ}=10\text{ h}\right)$ initially results in a transient oscillatory output response that returns to the stable equilibrium (Figure 2B, red) and eventually $\left(\text{τ}=15\text{ h}\right)$ generates an excitable response from a stable equilibrium that approximates a limited cycle orbit around an unstable equilibrium (Figure 2B, green). As is expected, the oscillatory behavior of the Goodwin oscillator is due to a sufficiently large maturation delay for a given repressor half-life [46].

To investigate the effect of the repressor’s half-life on the oscillatory behavior of the Goodwin topology, we first simulated the behavior of the mature repressor as a function of its half-life. The half-life of the repressor could be easily decreased experimentally through the addition of a repressor-specific NanoDeg (Figure 2C). The half-life of the repressor $\left(t_{1/2,\text{R}}\right)$ corresponding to the critical value for the Hopf bifurcation was found to be approximately 0.65 h. If the repressor’s half-life is lower than the critical value, the equilibrium of the repressor concentration is unstable, and the system is unbounded. Increasing the half-life of the repressor above the critical value (i.e., 0.67 h), results in a damped oscillation of the repressor concentration (Figure 2D, red). Increasing the degradation of the repressor using a repressor-specific NanoDeg with a half-life $\left(t_{1/2,\text{N}}\right)$ of 0.9 h, however, induces oscillations that arise from a sustained limited cycle around an unstable equilibrium (Figure 2D, green).

The NanoDeg synthesis rate window resulting in oscillatory behavior for the parameters used in this study is identified (32–52 nM·h^−1^). The oscillation amplitude (Figure 2E, left) and oscillation period (Figure 2E, right) increase linearly as a function of the NanoDeg synthesis rate until an upper bound is reached. A further increase in the NanoDeg’s expression results in excessive degradation of the repressor, lowering promoter repression and resulting in non-oscillating, constitutive expression of the repressor (Figure 2F).

These results demonstrate that NanoDeg-mediated control of a self-repressing protein produces an oscillatory system based on the Goodwin topology. The NanoDeg allows modulating the delay between the expression of the nascent repressor and the activity of the mature repressor. The amplitude and period of oscillation of the system are sensitive to the NanoDeg synthesis rate, indicating that modulation of the NanoDeg synthesis rate provides an additional method to experimentally tune the amplitude and period of a Goodwin oscillator.

### 3.3. Repressilator Regulation

**Figure 3.**
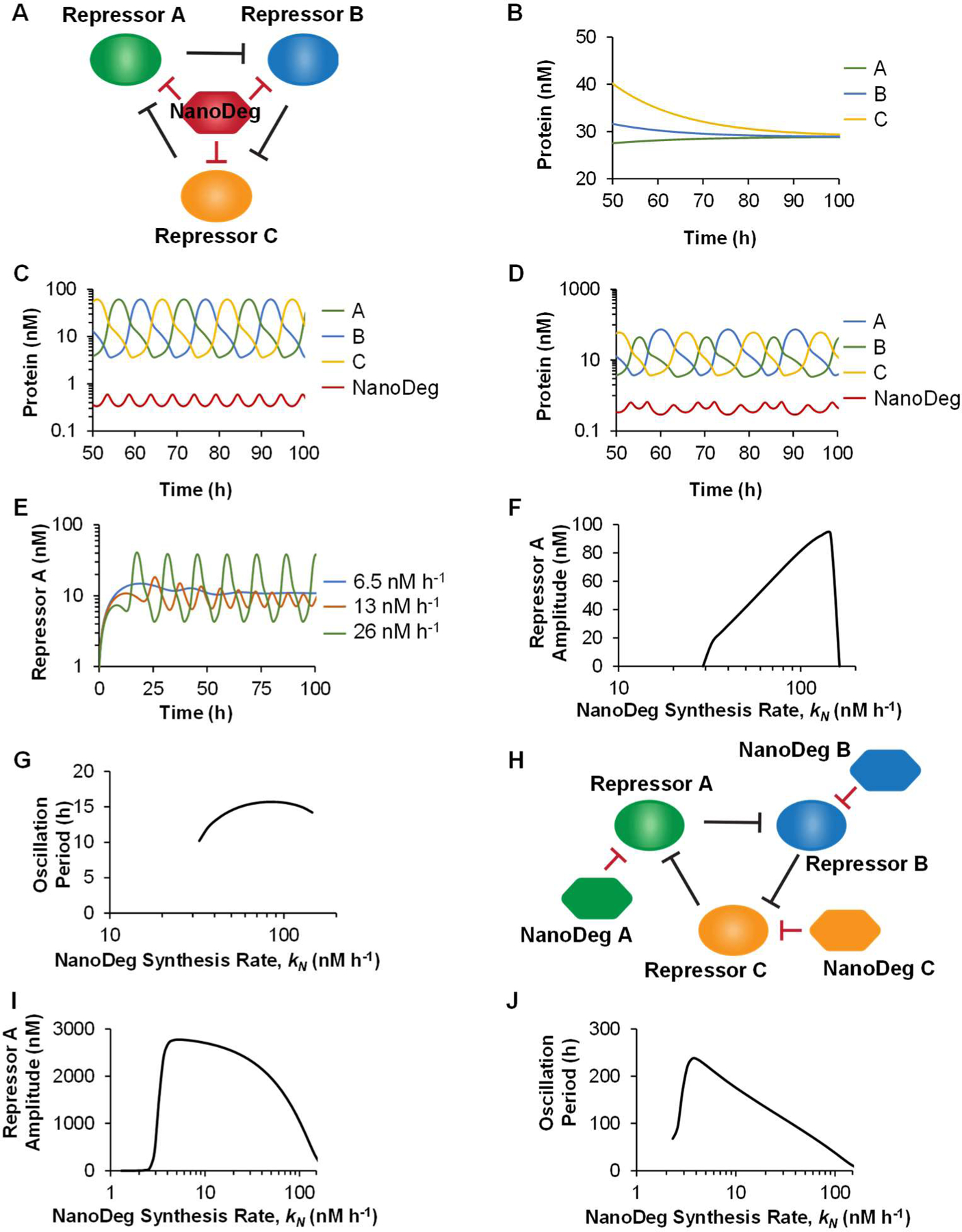
The repressilator. (**a**) Schematic representation of a three-node repressilator with a common NanoDeg targeting all three repressors. (**b**) Concentration of repressors in a three-node repressilator as a function of time. (**c**) Concentration of repressors and NanoDeg in a three-node repressilator with a common NanoDeg as a function of time. The three repressors are simulated using identical parameters. (**d**) Concentration of repressors and NanoDeg in a three-node repressilator with the expression of a common NanoDeg as a function of time. The three repressors are simulated using different synthesis rates: $k_{NA}=1\;\text{nM}\cdot {\text{h}}^{-1}$ (blue), $k_{NB}=10\;\text{nM}\cdot {\text{h}}^{-1}$ (green), and $k_{NC}=100\;\text{nM}\cdot {\text{h}}^{-1}$ (yellow). (**e**) Concentration of repressor A in a three-node repressilator with a common NanoDeg as a function of time and NanoDeg synthesis rate ($k_{\text{N}}=6.5\;\text{nM}\cdot {\text{h}}^{-1}$, blue; 13 nM·h^−1^, red; or 26 nM·h^−1^, green). (**f,g**) Amplitude (**f**) and period of oscillation (**g**) of a three-node repressilator with a common NanoDeg as a function of NanoDeg synthesis rate. (**h**) Schematic representation of a three-node repressilator with individual NanoDegs targeting each repressor. (**i,j**) Amplitude (**i**) and period of oscillation (**j**) of a three-node repressilator with individual NanoDegs targeting each repressor as a function of NanoDeg synthesis rate. The amplitude of oscillation was obtained by calculating the difference between the largest and lowest concentration of the reported protein in the region of oscillation using MATLAB’s “range” function. The period of oscillation was calculated by averaging the time interval between alternating zero crossings of the zero-mean trajectory in the region of oscillation.

Repressilators are genetic circuits consisting of repressors connected in series to generate a ring oscillator. Genetic repressilators were initially constructed in *E. coli* [45] and later identified in circadian clocks [47,48]. The features of repressilators that generate oscillatory outputs have been characterized [49–51]. Generally speaking, a system comprising an odd number of repressor nodes connected in a ring configuration resulting in at least one feedback loop presents oscillatory behavior, provided that each repressor node is sufficiently repressed by the corresponding repressor. Leaky expression from any repressor node may affect the oscillatory behavior of the output [51]. We investigated the use of the NanoDeg to mitigate leaky expression and generate oscillations in a non-oscillating repressilator topology. Specifically, we investigated two alternative methods to regulate the half-life of the repressors in a three-node repressilator topology that does not produce an oscillatory output due to the leaky expression of the three repressors. We first evaluated the use of a single NanoDeg that targeted each one of the three repressors, binding with the same affinity to each repressor and having the same effect on the repressors’ half-lives (Figure 3A). The repressilator’s components were simulated using the following equations:

(8) $$\frac{\text{d}A}{\;\text{d}t}=p_{A_{T}}\frac{k_{1}C^{r}\;+\;k_{2}K_{C}^{r}}{C^{r}\;+\;K_{C}^{r}}-\delta_{A}A-k_{on}AN\;+\;k_{off}T_{A}\frac{\text{d}B}{\;\text{d}t}=p_{B_{T}}\frac{k_{3}A^{m}\;+\;k_{4}K_{A}^{m}}{A^{m}\;+\;K_{A}^{m}}-\delta_{B}B-k_{on}BN\;+\;k_{off}T_{B}\frac{\text{d}C}{\;\text{d}t}\;\;\;\;\;\;\;=p_{C_{T}}\frac{k_{5}B^{n}\;+\;k_{6}K_{B}^{n}}{B^{n}\;+\;K_{B}^{n}}-\delta_{C}C-k_{on}CN\;+\;k_{off}T_{C}\frac{\text{d}N}{\;\text{d}t}\;\;\;\;\;\;\;=p_{N_{T}}k_{N}-\delta_{N}N-k_{on}(A\;+\;B\;+\;C)N\;+\;k_{off}\left(T_{A}\;+\;T_{B}\;+\;T_{C}\right)\frac{\text{d}T_{A}}{\;\text{d}t}=k_{on}AN\;+\;k_{off}T_{A}-\delta_{N}T_{A}\frac{\text{d}T_{B}}{\;\text{d}t}\;\;\;\;\;\;\;=k_{on}BN\;+\;k_{off}T_{B}-\delta_{N}T_{B}\frac{\text{d}T_{C}}{\;\text{d}t}=k_{on}CN\;+\;k_{off}T_{C}-\delta_{N}T_{C}$$

where A, B, and C are the concentrations of the three unbound repressors connected in series, N is the concentration of the unbound NanoDeg, $T_{A}$, $T_{B}$, and $T_{C}$ are the concentrations of the complexes formed upon association of the NanoDeg with Repressors A, B, and C, respectively. The expression of each repressor is simulated using Hill functions for repression by the corresponding repressor protein with Hill coefficients (m, n, and r), the minimum rate of synthesis due to repression ($k_{1}$, $k_{3}$, and $k_{5}$), the rate of synthesis due to leakiness ($k_{2}$, $k_{4}$, and $k_{6}$), and the equilibrium dissociation constant of each repressor bound to its operator sequence ($K_{A}$, $K_{B}$, and $K_{C}$). Constitutive expression of the NanoDeg is simulated using a constant synthesis rate $\left(k_{N}\right)$. Degradation of all protein species is simulated using linear degradation coefficients. The association and dissociation interactions between the repressors and the NanoDeg are modeled using a mass-action reaction model with association rate constant $k_{on}$ and dissociation rate constant $k_{off}$. The simulations were conducted using the parameter values reported in Table S3 of the Method Details unless otherwise specified.

Such an approach based on the use of a single NanoDeg that targets each one of the three repressors could be executed by developing a nanobody specific for a common repressor domain, such as the KRAB domain [52], or using a fluorescent protein-[38] or peptide tag-specific [39] nanobody upon co-expression of the repressors appropriately engineered by fusion to the fluorescent protein or peptide tag. We first modeled a repressilator topology that lacks expression of the NanoDeg and does not oscillate due to leaky expression of all three repressor nodes (Figure 3B). Oscillations in the repressilator are induced by the addition of a NanoDeg (half-life $t_{1/2,\text{N}}=0.9\;\text{h}$ and synthesis rate $k_{\text{N}}=23.1\text{ nM}\cdot {\text{h}}^{-1}$) that mediates the degradation of all three repressors (Figure 3C). Notably, because we assumed the three repressors present identical biological functionalities and were thus simulated using identical parameters, the concentration of the unbound NanoDeg also presents an oscillatory behavior with three times the frequency of each of the repressors and an oscillation amplitude inversely proportional to the amplitude of the repressors (Figure 3C). In a repressilator system based on repressors with different synthesis rates, the unbound NanoDeg still presents an oscillatory behavior with a frequency that is three times that of each repressor and an amplitude inversely proportional to the amplitude of the individual repressor being expressed (Figure 3D).

To investigate the effect of the NanoDeg synthesis rate on the period and amplitude of oscillation, we simulated the output of the repressilator based on repressors with the same synthesis rates upon modulation of the NanoDeg synthesis rate (Figure 3E). Suboptimal synthesis rate $\left(k_{\text{N}}\right)$ of the NanoDeg results in damped oscillations (Figure 3E, $k_{\text{N}}=6.5$ and 13 nM·h^−1^). Increasing the NanoDeg synthesis rate above a critical threshold results in sustained oscillatory behavior (Figure 3E, $k_{\text{N}}=26\;\text{nM}\cdot {\text{h}}^{-1}$). To further characterize the effect of the NanoDeg synthesis rate on the oscillatory features of a repressilator with a common NanoDeg, we analyzed the oscillation amplitude (Figure 3F) and oscillation period (Figure 3G) upon modulation of the NanoDeg synthesis rate within the range of NanoDeg synthesis rates that generates sustained oscillations ($k_{\text{N}}=23.1-145.7\;\text{nM}\cdot {\text{h}}^{-1}$). The lower bound of the NanoDeg synthesis rate interval corresponds to the minimum expression of NanoDeg required to counteract the effect of leakiness. Increasing the NanoDeg synthesis rate beyond the upper bound results in excessive degradation of at least one of the repressors so that the concentration required to repress its cognate promoter is never reached (Figure 3F,G). The oscillation amplitude increases substantially upon modulation of the NanoDeg synthesis rate within the range of NanoDeg synthesis rates producing oscillations (Figure 3F). The increase in oscillation amplitude observed upon an increase in NanoDeg-mediated degradation of the repressors is due to the reduced basal expression level of the repressors. The reduced basal expression level of repressors results in an increase in the dynamic range of each repressor’s concentration and, consequently, in the greater amplitude of oscillation (Figure 3F). In contrast, the oscillation period does not vary substantially upon modulation of the NanoDeg synthesis rate (Figure 3G).

We also investigated an alternative method to regulate the half-life of the repressors in a three-node repressilator topology that does not produce an oscillatory output due to leaky expression of the three repressors. Specifically, we tested the constitutive expression of three NanoDegs that are bound independently to the three repressors (Figure 3H). The system was simulated assuming the same binding affinity for each NanoDeg and the same effect on each repressor’s half-lives using the following equations:

(9) $$\frac{\text{d}A}{\;\text{d}t}=p_{A_{T}}\frac{k_{1}C^{r}\;+\;k_{2}K_{C}^{r}}{C^{r}\;+\;K_{C}^{r}}-\delta_{A}A-k_{on}AN_{A}\;+\;k_{off}T_{A}\frac{\text{d}B}{\;\text{d}t}=p_{B_{T}}\frac{k_{3}A^{m}\;+\;k_{4}K_{A}^{m}}{A^{m}\;+\;K_{A}^{m}}-\delta_{B}B-k_{on}BN_{B}\;+\;k_{off}T_{B}\frac{\text{d}C}{\;\text{d}t}\;\;\;\;\;\;\;=p_{C_{T}}\frac{k_{5}B^{n}\;+\;k_{6}K_{B}^{n}}{B^{n}\;+\;K_{B}^{n}}-\delta_{C}C-k_{on}CN_{C}\;+\;k_{off}T_{C}\frac{\text{d}N_{A}}{\;\text{d}t}=p_{N_{TA}}k_{NA}-\delta_{NA}N_{A}-k_{on}AN_{A}\;+\;k_{off}T_{A}\frac{\text{d}N_{B}}{\;\text{d}t}\;\;\;\;\;\;\;=p_{N_{TB}}k_{NB}-\delta_{NB}N_{B}-k_{on}AN_{B}\;+\;k_{off}T_{B}\frac{\text{d}N_{C}}{\;\text{d}t}=p_{N_{TC}}k_{NC}-\delta_{NC}N_{C}-k_{on}AN_{C}\;+\;k_{off}T_{C}\frac{\text{d}T_{A}}{\;\text{d}t}\;\;\;\;\;\;\;=k_{on}AN_{A}\;+\;k_{off}T_{A}-\delta_{NA}T_{A}\frac{\text{d}T_{B}}{\;\text{d}t}=k_{on}BN_{B}\;+\;k_{off}T_{B}-\delta_{NB}T_{B}\frac{\text{d}T_{C}}{\;\text{d}t}=k_{on}CN_{C}\;+\;k_{off}T_{C}-\delta_{NC}T_{C}$$

where A, B, and C are the concentrations of the three unbound repressors connected in series, $N_{A}$, $N_{B}$, and $N_{C}$ are the concentrations of the unbound NanoDegs that target A, B, and C, respectively, $T_{A}$, $T_{B}$, and $T_{C}$ are the concentrations of the complexes formed upon association of $N_{A}$, $N_{B}$, and $N_{C}$ with repressors A, B, and C, respectively. Each node of the repressilator is modeled with similar dynamics. The expression of each repressor is simulated using Hill functions for repression by the corresponding repressor protein with Hill coefficients (m, n, and r), the minimum rate of synthesis due to repression ($k_{1}$, $k_{3}$, and $k_{5}$), the rate of synthesis due to leakiness ($k_{2}$, $k_{4}$, and $k_{6}$), and the equilibrium dissociation constants of each repressor bound to its operator sequence ($K_{A}$, $K_{B}$, and $K_{C}$). Constitutive expression of $N_{A}$, $N_{B}$, and $N_{C}$ is simulated using constant synthesis rates ($k_{NA}$, $k_{NB}$, and $k_{NC}$). Degradation of all protein species is simulated using linear degradation coefficients. The association and dissociation interactions between the repressors and the NanoDegs are modeled using mass-action reaction models with an association rate constant $k_{on}$ and dissociation rate constant $k_{off}$. The simulations are conducted using the parameter values reported in Table S4 of the Method Details unless the otherwise specified.

Similar to the use of a common NanoDeg, the use of independent NanoDegs also induced oscillation by counteracting the effect of the leaky expression of the repressors. Both the oscillation amplitude and period, however, were sensitive to the NanoDeg synthesis rate (Figure 3I,J). The oscillation amplitude increased rapidly at the onset of oscillation as a result of the NanoDegs decreasing the basal concentration of the repressors and, consequently, increasing the dynamic range of each repressor’s concentration (Figure 3I). As the NanoDeg synthesis rate increased above a critical value ($k_{\text{N}}=3.7\;{\text{nM·h}}^{-1}$), the oscillation amplitude decreased upon modulation of the NanoDeg synthesis rate within the range of NanoDeg synthesis rates producing oscillations (Figure 3I). The oscillation period decreased upon increasing NanoDeg synthesis rate as individual NanoDegs result in more rapid degradation of each repressor and thus faster transitions between expression of each repressilator node (Figure 3J). The different features of the oscillation period of the system expressing a common NanoDeg and the system expressing individual NanoDegs (compare Figure 3G and Figure 3J) suggest that the integration of a common NanoDeg produces secondary coupling [53] between the repressor nodes in the repressilator system.

These results demonstrate the use of the NanoDeg to modulate the oscillatory behavior of a three-node repressilator. The use of a common NanoDeg targeting all three repressors allows modulating oscillation amplitude without impacting dramatically the oscillation period. The use of three repressor-specific NanoDegs, on the other hand, results in the modulation of both the oscillation amplitude and oscillation period of the repressilator.

### 3.4. NanoDeg Repressilator

**Figure 4.**
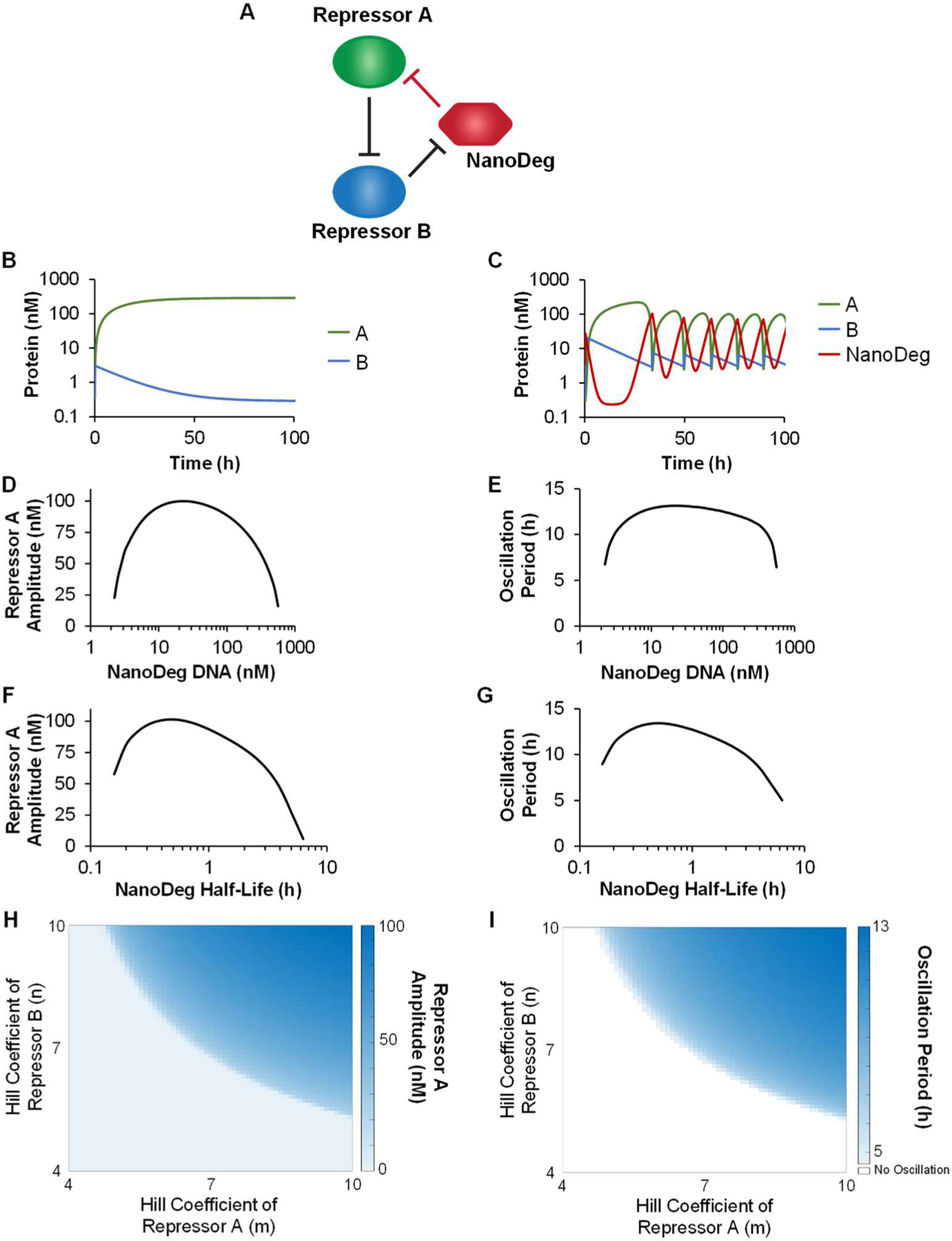
The NanoDeg repressilator. (**a**) Schematic representation of a mixed-mode repressilator based on the integration of a NanoDeg into a network of two repressors in series. (**b**) Concentration of repressors in a network of two repressors in series as a function of time. (**c**) Concentration of repressors and NanoDeg in the mixed-mode repressilator based on the integration of a NanoDeg into a network of two repressors in series as a function of time. (**d,e**) Amplitude (**d**) and period of oscillation (**e**) of Repressor A in the mixed-mode NanoDeg repressilator as a function of the concentration of DNA encoding the NanoDeg gene. (**f,g**) Amplitude (**f**) and period of oscillation (**g**) of Repressor A in the mixed-mode NanoDeg repressilator as a function of NanoDeg half-life. (**h,i**) Amplitude (**h**) and period of oscillation (**i**) of Repressor A in the mixed mode NanoDeg repressilator of Hill coefficients of Repressor A (m) and Repressor B (n). The amplitude of oscillation was obtained by calculating the difference between the largest and lowest concentration of the reported protein in the region of oscillation using MATLAB’s “range” function. The period of oscillation was calculated by averaging the time interval between alternating zero crossings of the zero-mean trajectory in the region of oscillation.

We investigated the design of a mixed-mode repressilator circuit based on the integration of a NanoDeg into an existing network of two repressors in series [54–57]. Specifically, we investigated the design of a three-node repressilator consisting of two transcriptional regulators (Repressors A and B) and a post-translational regulator (the NanoDeg) (Figure 4A). Similar to the other repressilator topologies, this system was simulated using a model based on ordinary differential equations describing the concentration of the species involved as detailed in the Methods section. Repressor A was modeled as controlled by a constant synthesis rate and degradation rate depending on the interaction with the NanoDeg, which was in turn modeled based on mass action expressions. Repressor B and the NanoDeg were modeled as controlled by synthesis rates following Hill functions for a repressor (Repressor A and Repressor B, respectively) and by constant degradation rates.

The NanoDeg repressilator was simulated using the following equations:

(10) $$\frac{\text{d}A}{\;\text{d}t}=p_{A_{T}}k_{1}-\delta_{A}A-k_{on}AN\;+\;k_{off}C\frac{\text{d}B}{\;\text{d}t}=p_{B_{T}}\frac{k_{2}A^{m}\;+\;k_{3}K_{A}^{m}}{A^{m}\;+\;K_{A}^{m}}-\delta_{B}B\frac{\text{d}N}{\;\text{d}t}\;\;\;\;\;\;\;=p_{N_{T}}\frac{k_{4}B^{n}\;+\;k_{5}K_{B}^{n}}{B^{n}\;+\;K_{B}^{n}}-\delta_{N}N-k_{on}AN\;+\;k_{off}C\frac{\text{d}C}{\;\text{d}t}=k_{on}AN-k_{off}C-\delta_{N}C$$

where A is the concentration of the unbound Repressor A, B is the concentration of unbound Repressor B, N is the concentration of the unbound NanoDeg, and C is the concentration of the complex formed upon association of the NanoDeg with the repressor A. Constitutive expression of Repressor A is modeled using a constant synthesis rate ($k_{1}$). The expression of the Repressor B and the NanoDeg are simulated using a Hill function of repression by Repressor A and Repressor B, respectively, with Hill coefficients (m and n), the minimum rate of synthesis due to repression ($k_{2}$ and $k_{4}$), the rate of synthesis due to leakiness ($k_{3}$ and $k_{5}$), and the equilibrium dissociation constant of each repressor bound to its operator sequence ($K_{A}$ and $K_{B}$). The degradation of all proteins is simulated using linear degradation coefficients. The association and dissociation interactions between repressor A and the NanoDeg are modeled using a mass-action reaction model with the association rate constant $k_{on}$ and a dissociation rate constant $k_{off}$. The simulations are conducted using the parameter values reported in Table S5 of the Method Details unless the otherwise specified.

Simulations revealed that the network based on two stable repressors ($t_{1/2}=11\;\text{h}$) in series and lacking the NanoDeg does not oscillate (Figure 4B). Integrating the NanoDeg as a third node linked to the two repressors into a ring configuration results in a topology similar to that of a three-node repressilator (Figure 3A), with the significant distinction that the interaction between the third node (the NanoDeg) and the first node (Repressor A) is regulated at the post-translational rather than transcriptional level. Oscillatory behavior is observed upon the addition of a NanoDeg exhibiting a half-life ($t_{1/2,\text{N}}=0.9\;\text{h}$) that results in sufficient depletion of the Repressor A levels (Figure 4C).

To investigate the design rules of a mixed-mode repressilator circuit, we simulated the output’s amplitude and period of oscillation as a function of the concentration of DNA encoding the NanoDeg, the NanoDeg half-life, and the sensitivity (Hill coefficients) of Repressor A and Repressor B. For the simulated system, we identified the range of NanoDeg-encoding DNA resulting in oscillatory behavior (Figure 4D,E) and ranging from the minimum value (2 nM) corresponding to the NanoDeg levels needed to achieve sufficient levels of Repressor A degradation to the maximum value (562 nM) corresponding to NanoDeg levels leading to excessive degradation of Repressor A. The oscillation amplitude was found to be sensitive to the concentration of NanoDeg-encoding DNA and reached a maximum at an intermediate concentration (22.4 nM, Figure 4D). The oscillation period was found to be sensitive to variations in NanoDeg-encoding DNA near the limits of the DNA concentration range where oscillations occur but otherwise did not vary significantly with variation in the DNA concentration (Figure 4E).

We also identified the range of NanoDeg half-life $\left(t_{1/2,\text{N}}\right)$ resulting in oscillatory behavior. We found that the oscillation amplitude (Figure 4F) and period (Figure 4G) increased as a function of NanoDeg half-life until a maximum time ($t_{1/2,\text{N}}=0.5\;\text{h}$). A further increase in the NanoDeg half-life resulted in a decrease in both the amplitude and period of oscillations. As the half-life of the NanoDeg approached that of the repressors controlling the first and second node of the circuit, the oscillation amplitude and period were reduced, and the network transitioned to a stable equilibrium dominated by expression of Repressor A due to a lack of NanoDeg-mediated depletion of Repressor A (Figure 4F,G).

Simulations of this NanoDeg repressilator topology were conducted using sensitivities of Repressor A (Hill coefficient m) and Repressor B (Hill coefficient n) that result in an oscillatory output (m=10, n=10) [58]. Repressilators based on nodes controlled by repressors presenting high sensitivity, as defined by the corresponding Hill coefficients, are more likely to oscillate [50]. To investigate the impact of the sensitivity of Repressor A and Repressor B on the NanoDeg repressilator oscillatory behavior, we analyzed the amplitude and the period of oscillation of the NanoDeg repressilator as a function of pairs of Hill coefficients (m, n) and identified the range of Hill coefficient values that results in oscillatory behavior (Figure 4H,I). Of note, it appears that the NanoDeg repressilator tolerates lower sensitivity of the Repressor A (m>4.8) than of the Repressor B (n>5.4). Furthermore, we observed robust amplitude (Figure 4H) and period (Figure 4I) of oscillations within the range of sensitivity of Repressor A and Repressor B where oscillations occur.

These results demonstrate the use of the NanoDeg to generate oscillator topologies conventionally implemented using transcriptional repressors in series. Such a strategy might be limited to the design of circuits in which the NanoDeg is linked to repressor pairs that present particularly high sensitivities, such as MAPK pathway repressors presenting switch-like dynamics [58–60].

## 4. Discussion

Oscillatory behaviors in biological systems are typically investigated by constructing isolated synthetic gene networks in cells and monitoring their behavior [45,47,61–63], or by monitoring native cellular systems [5,48,64–66]. Building genetic circuits orthogonal to the cellular circuitry is a powerful strategy for investigating the design rules of biological systems with periodic behavior but is also remarkably challenging to implement experimentally, pointing to a critical need for efficient tools to perturb the behavior of existing oscillatory systems by altering the levels of key components of the underlying regulatory network.

Perturbations of native oscillatory systems are commonly achieved using chemical genetics approaches mainly based on the use of small molecule inhibitors. This strategy has enabled investigations of many fundamental cellular pathways, including the p53 network, which was perturbed using an Mdm2 inhibitor [5] and of the Notch oscillatory signaling system, which was perturbed using an inhibitor of Notch cleavage [66]. While characterized by easy, efficient, and rapid delivery, dose-dependence, and reversibility, small molecule-based approaches are often plagued by low specificity, off-target effects, target-dependent efficiency, and lack of temporal control. Chemical genetics tools are also typically dependent on slow lead identification and optimization processes.

An alternative approach to investigating biological oscillators relies on the genetic manipulation of a network’s components, mainly through the generation of genetic knockouts [48] or variants with desired functional properties [65]. Such approaches are restricted to applications in which cellular viability is not affected by the genetic modifications needed to achieve the desired network perturbation. Moreover, network perturbations achieved via genetic manipulations of circuit components are typically not compatible with quantitative control over the component’s levels or properties.

A few additional approaches have emerged, including strategies based on siRNA-mediated regulation [67] and post-translational modulation based on DNA-protein interactions [18,68]. The effects of such network modifications, however, are limited to target sequestration [42] or timescale effects due to retroactivity [40,69].

In the present study, we investigate the post-translational modulation of network components as a strategy to generate oscillatory systems and modulate the features of oscillatory behaviors. Post-translational control is achieved by tuning the target degradation rate via nanobody-mediated depletion using the NanoDeg platform. Simulations of the behavior of different oscillatory topologies provide an extensive characterization of the role of post-translational regulation in the design of genetic oscillators. Specifically, the NanoDeg can be used to achieve the separation of timescales of circuit components required to generate an activator-repressor oscillator and a Goodwin oscillator. We demonstrate the use of the NanoDeg to mitigate leaky repression that sometimes dampens oscillation in Goodwin oscillator and repressilator topologies. We also propose the insights to integrate the NanoDeg within a repressor series to generate a mixed repressilator based on nodes generating transcriptional and post-translational feedback.

The results of this study provide guidelines to the use of the NanoDeg as a universal platform for post-translational perturbation of native and synthetic networks that results in quantitative control of the network oscillatory behavior. These guidelines can be implemented experimentally due to the plug-and-play nature of the NanoDeg platform. The nanobody, which provides target recognition, is the smallest monomeric antigen-binding element derived from a functional antibody and presents a high solubility, low aggregation propensity [70], small size that allows tissue penetration and recognition of hidden epitopes [71], and the ability to recognize conformational epitopes [72] and conformational intermediates [73]. High affinity nanobodies can be readily isolated from immunized [74–77], naïve [78,79], and synthetic libraries [80,81] through the use of protein displays technologies. The NanoDeg can be thus adapted to target virtually any cellular protein, thus providing a versatile tool to generate and modulate a wide range of oscillatory systems without requirement for burdensome target manipulation. The degron moiety of the NanoDeg can be customized with respect to the rate and mechanism of degradation which enable the control of the degradation rate of the network component with exquisite precision and through different ubiquitin-dependent as well as ubiquitin-independent pathways for proteasomal degradation. Importantly, degron-mediated depletion relies on a diverse repertoire of sequences for tunable, reversible, and even orthogonal controls over the degradation [23–26,82,83]. As a result, the degradation-signaling unit of the NanoDeg system could be engineered to modulate the levels of the network components that generate the desired oscillatory behavior.

## Supplementary Material

Supplementary Information
