# Supplementary material for: Design of Oscillatory Networks through Post-Translational Control of Network Components
Source: Synth Biol Eng. Author manuscript; Available in PMC 2024 Apr 8. (PMC11000592; doi:10.35534/sbe.2023.10004)
Supplement: Supplementary Information [file NIHMS1973587-supplement-Supplementary_Information.pdf]

SCIEPublish

Supplementary Information

Design of Oscillatory Networks through Post-translational Control of Network Components

Brianna E.K. Jayanthi [1], Shridhar Jayanthi [2] and Laura Segatori [1,2,3,4,*]

[1] Systems, Synthetic, and Physical Biology Graduate Program, Rice University, Houston, TX 77005, USA
[2] Department of Bioengineering, Rice University, Houston, Texas 77005, USA
[3] Department of Chemical & Biomolecular Engineering, Rice University, Houston, Texas 77005, USA
[4] Department of BioSciences, Rice University, Houston, Texas 77005, USA
* Corresponding author. E-mail: segatori@rice.edu (L.S.)

Table S1. Parameters used in activator-repressor simulations.

Parameter	Description	Value	Source
p_{A_T}	Activator gene DNA concentration	1 nM	This work
p_{B_T}	Repressor gene DNA concentration	1 nM	This work
p_{N_T}	NanoDeg gene DNA concentration	1 nM	This work
K_A	Activator-operator equilibrium dissociation constant	3 nM	[1]
K_B	Repressor-operator equilibrium dissociation constant	3 nM	[1]
δ_A	Activator degradation rate	4 h	This work
δ_B	Repressor degradation rate	4 h	This work
δ_N	NanoDeg degradation rate	0.9 h	[1]
α_1	Activator synthesis rate with maximum self-activation	112.5 h^{-1}	[2]
α_2	Activator synthesis rate with leaky self-activation	1 h^{-1}	Modified from [2]
β_1	Activator synthesis rate with maximum repression	0.04 h^{-1}	Modified from [2]
β_2	Activator synthesis rate with leaky repression	1.8 h^{-1}	[2]
m	Activator Hill coefficient	2	This work
n	Repressor Hill coefficient	2	This work
k_5	Repressor synthesis rate with maximum activation	36 h^{-1}	[2]
k_6	Repressor synthesis rate with leaky activation	0.05 h^{-1}	Modified from [2]
k_{on}	NanoDeg-activator association rate constant	0.6264 nM h^{-1}	[1]
k_{off}	NanoDeg-activator dissociation rate constant	2.7648 h^{-1}	[1]
k_N	NanoDeg synthesis rate	5 h^{-1}	This work

Table S2. Parameters used in Goodwin oscillator simulations.

Parameter	Description	Value	Source
p_{A_T}	Repressor gene DNA concentration	1 nM	This work
p_{N_T}	NanoDeg gene DNA concentration	1 nM	This work
K_A	Repressor-operator equilibrium dissociation constant	3nM	[2]
δ_A	Repressor degradation rate	11 h	This work
δ_N	NanoDeg degradation rate	0.9 h	[1]
β_1	Repressor synthesis rate with maximum repression	1.8 h^{-1}	[2]
β_2	Repressor synthesis rate with leaky repression	181 h^{-1}	[2]
m	Repressor Hill coefficient	2	This work
k_{on}	NanoDeg-mature repressor association rate constant	0.6264 nM h^{-1}	[1]
k_{off}	NanoDeg-mature repressor dissociation rate constant	2.7648 h^{-1}	[1]
k_N	NanoDeg synthesis rate	5 nM/h	This work
τ	Repressor maturation time	0.5 h	This work

https://doi.org/10.35534/sbe.2023.10004

Sable S3. Repressilator with a common NanoDeg.

Parameter	Description	Value	Source
p_{A_T}	Repressor A gene DNA concentration	1 nM	This work
p_{B_T}	Repressor B gene DNA concentration	1 nM	This work
p_{C_T}	Repressor C gene DNA concentration	1 nM	This work
p_{N_T}	NanoDeg gene DNA concentration	1 nM	This work
K_A	Repressor A-operator equilibrium dissociation constant	3 nM	[2]
K_B	Repressor B-operator equilibrium dissociation constant	3 nM	[2]
K_C	Repressor C-operator equilibrium dissociation constant	3 nM	[2]
δ_A	Repressor A degradation rate	11 h	This work
δ_B	Repressor B degradation rate	11 h	This work
δ_C	Repressor C degradation rate	11 h	This work
δ_N	NanoDeg degradation rate	0.9 h	[1]
k_1	Repressor A synthesis rate with maximum repression	1.8 h^{-1}	[2]
k_2	Repressor A synthesis rate with leaky repression	181 h^{-1}	[2]
k_3	Repressor B synthesis rate with maximum repression	1.8 h^{-1}	[2]
k_4	Repressor B synthesis rate with leaky repression	181 h^{-1}	[2]
k_5	Repressor C synthesis rate with maximum repression	1.8 h^{-1}	[2]
k_6	Repressor C synthesis rate with leaky repression.	181 h^{-1}	[2]
m	Repressor A Hill coefficient	4	This work
n	Repressor B Hill coefficient	4	This work
r	Repressor C Hill coefficient	4	This work
k_{on}	NanoDeg-repressor association rate constant	0.6264 nM h^{-1}	[1]
k_{off}	NanoDeg-repressor dissociation rate constant	2.7648 h^{-1}	[1]
k_N	NanoDeg synthesis rate	6.5 nM/h	This work

Table S4. Parameters used in Repressilator with individual NanoDegs simulations.

Parameter	Description	Value	Source
p_{A_T}	Repressor A gene DNA concentration	1 nM	This work
p_{B_T}	Repressor B gene DNA concentration	1 nM	This work
p_{C_T}	Repressor A gene DNA concentration	1 nM	This work
$p_{N_{TA}}$	NanoDeg A gene DNA concentration	1 nM	This work
$p_{N_{TB}}$	NanoDeg B gene DNA concentration	1 nM	This work
$p_{N_{TC}}$	NanoDeg C gene DNA concentration	1 nM	This work
K_A	Repressor A-operator equilibrium dissociation constant	3 nM	[2]
K_B	Repressor B-operator equilibrium dissociation constant	3nM	[2]
K_B	Repressor C-operator equilibrium dissociation constant	3nM	[2]
δ_A	Repressor A degradation rate	11 h	This work
δ_B	Repressor B degradation rate	11 h	This work
δ_C	Repressor C degradation rate	11 h	This work
δ_N	NanoDeg degradation rate	0.9 h	[1]
k_1	Repressor A synthesis rate with maximum repression	1.8 h^{-1}	[2]
k_2	Repressor A synthesis rate with leaky repression	181 h^{-1}	[2]
k_3	Repressor B synthesis rate with maximum repression	1.8 h^{-1}	[2]
k_4	Repressor B synthesis rate with leaky repression	181 h^{-1}	[2]
k_5	Repressor C synthesis rate with maximum repression	1.8 h^{-1}	[2]
k_6	Repressor C synthesis rate with leaky repression	181 h^{-1}	[2]
m	Repressor A Hill coefficient	4	This work
n	Repressor B Hill coefficient	4	This work
r	Repressor C Hill coefficient	4	This work
k_{on}	NanoDeg-repressor association rate constant	0.6264 nM h^{-1}	[1]
k_{off}	NanoDeg-repressor dissociation rate constant	2.7648 h^{-1}	[1]
k_{NA}	NanoDeg A synthesis rate	5 nM/h	This work
k_{NB}	NanoDeg B synthesis rate	5 nM/h	This work

k_{NC}	NanoDeg C synthesis rate	5 nM/h	This work

Table S5. Parameters used in NanoDeg repressilator simulations.

**Parameter**	**Description**	**Value**	**Source**
p_{A_T}	Repressor A gene DNA concentration	1 nM	This work
p_{B_T}	Repressor B gene DNA concentration	1 nM	This work
p_{N_T}	NanoDeg gene DNA concentration	1 nM	This work
K_A	Repressor A-operator equilibrium dissociation constant	3 nM	[2]
K_B	Repressor B-operator equilibrium dissociation constant	3 nM	[2]
δ_A	Repressor A degradation rate	11 h	This work
δ_B	Repressor B degradation rate	11 h	This work
δ_N	NanoDeg degradation rate	0.9 h	[1]
k_1	Repressor A synthesis rate	18.1 h^{-1}	This work
k_2	Repressor B synthesis rate with maximum repression	0.018 h^{-1}	Modified from [2]
k_3	Repressor B synthesis rate with leaky repression	18.1 h^{-1}	Modified from [2]
k_4	NanoDeg synthesis rate with maximum repression	0.018 h^{-1}	Modified from [2]
k_5	NanoDeg synthesis rate with leaky repression	18.1 h^{-1}	Modified from [2]
m	Repressor A Hill coefficient	10	This work
n	Repressor B Hill coefficient	10	This work
k_{on}	NanoDeg-Repressor A association rate constant	0.6264 nM h^{-1}	[1]
k_{off}	NanoDeg-Repressor A dissociation rate constant	2.7648 h^{-1}	[1]

References

1. Zhao, W.; Pferdehirt, L.; Segatori, L. Quantitatively Predictable Control of Cellular Protein Levels through Proteasomal Degradation. *ACS Synth. Biol.* **2018**, *7*, 540–552.
2. Zhao, W.; Bonem, M.; McWhite, C.; Silberg, J.J.; Segatori, L. Sensitive detection of proteasomal activation using the Deg-On mammalian synthetic gene circuit. *Nat. Commun.* **2014**, *5*, 3612.
